# Digital Health Technologies in Professional Healthcare Training: A Conceptual Framework for AI-Based Adaptive Clinical Simulation and Health Communication

**DOI:** 10.3390/healthcare14213643

**Published:** 2026-10-27

**Authors:** Syed Uzair Jaffri, Ah-Choo Koo, Choo-Yee Ting, Salman Hussain

**Affiliations:** 1 Faculty of Creative Multimedia, Multimedia University, Cyberjaya 63100, Malaysia; syed.uzair.jaffri@student.mmu.edu.my; 2 Centre for Interaction and Experience Design, CoE of Immersive Experience, Multimedia University, Cyberjaya 63100, Malaysia; 3 Centre for Big Data and Blockchain Technologies, CoE of Advanced Cloud, Multimedia University, Cyberjaya 63100, Malaysia; cyting@mmu.edu.my; 4 Faculty of Business Administration, Iqra University, Karachi 75850, Pakistan; salman.hussain@iqra.edu.pk

**Keywords:** digital health technologies, artificial intelligence, healthcare training, clinical simulation, health communication, adaptive learning, affective computing, learning analytics, virtual patients, competency-based education

## Abstract

Digital health technologies are reshaping professional healthcare training by enabling more adaptable approaches to clinical simulation, health communication practice, learner assessment, and adaptive learning. However, existing approaches often address these skills separately, with limited integration of clinical reasoning, competence-focused assessment, dynamic simulated-patient responses, patient-centered communication, and adaptive feedback within a single learning cycle. This article presents an evidence-informed conceptual framework for AI-based adaptive clinical simulation and health communication in healthcare training. A structured literature review and conceptual synthesis were used to identify educational requirements and design implications across six domains: clinical reasoning, generative artificial intelligence, simulation and virtual patients, patient-centered communication and empathy, competency-oriented assessment and adaptive learning, and safety and governance. The proposed framework incorporates seven design requirements within an 11-stage closed loop linking learner interaction, behavior capture, simulated-patient state updating, assessment, adaptive response, performance analytics, feedback, and scenario progression. The simulated patient is represented through interconnected clinical, informational, affective, and interactional states, allowing learner behavior to influence subsequent information disclosure and interaction within predefined educational and safety boundaries. Competency standards, clinical guardrails, escalation rules, and high-stakes decisions remain under educator and institutional governance. The framework is proposed as a conceptual design model rather than a validated educational intervention. Its value therefore requires the staged evaluation of technical consistency, assessment validity, safety, educational effectiveness, retention, and behavioral transfer before claims regarding educational or clinical outcomes can be established.

## 1. Introduction

### 1.1. Digital Transformation in Healthcare Training

Digital technologies are increasingly becoming a part of clinical practice. The adoption of electronic health records (EHRs), telehealth, clinical decision support systems, data-driven care, mobile health apps, and artificial intelligence (AI) impacts the way that professionals access, interpret, communicate, and act on clinical information. Thus, training must not only impart knowledge and procedural skills to learners, but also clinical reasoning, communication and collaboration, and decision-making skills in technology-mediated environments [1,2,3].

For repeated practice in a controlled setting, simulation is a suitable educational tool to allow learners to practice specific competencies, provide feedback, and learn from mistakes without putting patients at unnecessary risk. Simulation is not just about fidelity; it is also about purposeful design, clear goals, deliberate practice, progressive challenge, feedback, and curricular integration [1,2]. Virtual patients further these concepts by offering a structured opportunity to practice clinical information gathering, reasoning, and communication in a low-risk environment. Evidence from systematic reviews supports their educational potential, although substantial heterogeneity remains in case design, implementation, outcomes, and study quality [4].

Generative AI is another opportunity to create more flexible and responsive simulated encounters. Generative AI can facilitate open-ended interaction, where learners ask questions, explain things, and make decisions in their own words, not just in the limited branches or answer options provided. There is recent evidence of the increasing use of generative-AI-supported virtual patients in health professions education, with positive results regarding feasibility and selected learning outcomes, and significant variation in design, evaluation, educational foundation, and interactional complexity [5,6]. The most recent systematic review of virtual patients supported by GenAI also highlighted the continued lack of emotional-behavioral complexity, adaptability, theoretical support, standardized design, and longitudinal rigorous evaluation [5].

### 1.2. AI-Based Simulation and Virtual Patients

Conventional virtual patients use a structured case, branching logic, or predefined responses. Generative AI can enhance interactional flexibility, enabling learners to ask open-ended questions and receive context-sensitive responses [4,5,6]. Generative-AI-based virtual patients are increasingly used in the health professions. Research suggests that generative AI can support health professions’ education, although the evidence remains heterogeneous and key limitations persist in adaptability, emotional-behavioral complexity, educational grounding, and evaluation [5,6].

The main educational challenge, therefore, is not simply the ability of an AI system to generate fluent dialog. The responses from AI should be consistent with the clinical scenario, simulated-patient state, learner behavior, and intended competency. If this is not integrated, then the responses may be plausible but not educationally appropriate or not in line with the encounter. This underscores the importance of an architecture that integrates AI-generated interactions with clinical reasoning, communication, competency-based assessment, and adaptive feedback.

### 1.3. The Integration Gap

Current studies address the elements of AI-supported clinical training separately: simulation and deliberate practice with feedback [1,2]; virtual patients for clinical reasoning and information gathering [4]; flexible interaction with generative AI [5,6]; patient-centered communication and observable communication and empathy-related behaviors [7,8]; competency-based education that links performance to defined competencies [9]; and AI assessment and governance, including validity, safety, and human oversight [10,11].

The key challenge is the ability to combine these features into a single adaptive learning loop. Existing approaches have yet to establish an architecture that sufficiently links persistent simulated-patient state, communication, learner behavior, competency assessment, clinical reasoning, adaptive feedback, and governance. This article does not introduce a new individual competency for AI, but rather a conceptual framework that brings these elements together in a coordinated way, based on evidence, and around a simulated clinical encounter that evolves.

### 1.4. Aim and Contribution

This article suggests an evidence-informed conceptual model for the use of AI for adaptive clinical simulation and health communication in professional healthcare training. It combines elements of simulation-based education, virtual patients, generative AI, patient-centered communication, adaptive learning, learning analytics, and AI governance into a coherent structure.

The purpose of this framework is to be conceptual, rather than to represent a new AI technology or to illustrate the effectiveness of education. Rather, it describes the ways in which clinical context, dynamic simulated-patient state, learner behavior, reasoning, communication, adaptation, assessment, feedback, and governance can be integrated into a single training context. Three questions guide the synthesis: What requirements identified in the literature should the architecture support? How can these requirements be integrated into a coherent architecture? How should the architecture be validated before claims are made regarding educational or clinical effectiveness?

## 2. Methods

### 2.1. Review Design

This article adopts a structured narrative review and conceptual-synthesis method to pinpoint the educational, clinical, and technological needs for adaptive clinical simulation and health communication using AI in professional healthcare education. We have selected this approach to integrate a variety of evidence from health professions’ education, clinical simulation, virtual patients, generative artificial intelligence (AI), patient-centered communication, competency-based education, adaptive learning, assessment, and AI governance, and to translate this into a coherent conceptual architecture. The review is conceptually organized around the evidence domains and design questions, and does not make stronger claims that would necessitate a systematic review, formal risk-of-bias assessment, or meta-analysis [12]. The intent was not to measure an overall intervention effect or to determine the relative effectiveness of current technologies.

The resulting framework is not a proven educational intervention or evidence that an integrated AI environment is better than current approaches, but rather a conceptual architecture based on evidence.

### 2.2. Evidence Identification and Selection

The evidence identification search process was iterative and domain-based, using PubMed and the relevant publisher and authoritative organizational literature. The six broad search concept areas were artificial intelligence and generative AI; health professions education and competency-based education; clinical simulation and virtual patients; patient-centered communication and empathy; adaptive learning, assessment, and learning analytics; and AI safety, ethics, and governance. The recent literature on AI was prioritized, while retaining seminal educational and simulation studies still relevant to the proposed architecture. Representative search combinations included AI with education and simulation terms (e.g., generative AI/large language model with health professions education; virtual patient/clinical simulation with communication, empathy, or reasoning; and adaptive learning/learning analytics with assessment).

Sources that contributed to at least one of four purposes within the conceptual synthesis were retained: (i) educational principle; (ii) design requirement identification; (iii) identifying a limitation, risk, or governance condition; or (iv) validation evidence. Importance was given to systematic reviews of virtual patients, the literature on clinical simulation, consensus or guidance documents, meta-analyses, framework-development studies, and AI in health professions education.

Because it was a structured narrative review, the source set was assembled for conceptual relevance and design utility rather than for exhaustive statistical aggregation. The sources were then organized by their contribution to the six evidence domains described below.

Search dates, complete search strings, and the numbers of records identified, screened, and excluded were not recorded during this iterative search. They are therefore not reported, and no flow diagram is presented, rather than being reconstructed retrospectively. What can be reported is the composition of the resulting evidence base. The final set comprises 38 references published between 2005 and 2026: 11 systematic reviews or meta-analyses, six scoping, critical, or comparative reviews, two guidance documents, nine conceptual, methodological, policy, or validation-framework papers, and ten primary empirical or technical studies. Targeted searches on assessment validation and on intelligent tutoring and adaptive-simulation comparators (Section 6.1) supplemented the initial domain-based search.

Evidence identification, selection, and categorization were performed by one author without independent duplicate screening. To mitigate this limitation, each framework element is labeled by provenance (Section 2.3), and the supporting references for each design requirement are provided in Section 3.9. While this approach enhances transparency, it does not render the search reproducible (Section 9.4).

### 2.3. Conceptual Synthesis

The evidence was categorized into six areas: (1) simulation and virtual patients; (2) clinical reasoning; (3) generative AI; (4) patient-centered communication and empathy; (5) competency-oriented assessment and adaptive learning; and (6) safety and governance. The synthesis used these domains to identify recurring educational needs, capabilities, limitations, and safety conditions.

The proposed architecture is conceptual, and the synthesis does not state that the system has been empirically validated. The synthesis was based on the logic of need, requirement, capability, and feedback loop. Educational needs and limitations were translated into explicit design requirements, which were mapped to capabilities within the proposed architecture, and then those capabilities were linked through a recursive learner–patient–assessment cycle in which defined competencies interpret performance, learner behavior influences the simulated encounter, and feedback informs subsequent learning opportunities. This process is based on the conceptual-framework development approach, with the claims of the conceptual framework being linked to the evidence [13].

Three levels of provenance were identified and included in the proposed framework when translating the evidence. Literature-derived elements are educational principles, findings, limitations, or governance conditions that are directly supported by the reviewed sources. Synthesis-derived elements are relationships or implications that are drawn by synthesizing evidence across the six domains. The author-proposed design elements are the architectural decisions that were made to operationalize the synthesis, such as the organization of the seven design requirements, the four simulated-patient state dimensions, and the 11-stage closed-loop architecture.

This distinction was kept to prevent conceptual design decisions from appearing as empirical results. The resulting architecture is thus a conceptual synthesis based on evidence, and the elements of this architecture must be tested empirically before claims can be made about educational effectiveness, behavioral transfer, or clinical outcomes. The relationships between evidence and design are explicitly stated in the following traceability analysis.

## 3. Theoretical Foundation and Evidence Synthesis

### 3.1. Theoretical Foundation

The framework does not introduce a new learning theory but rather builds on the existing principles of simulation-based education, virtual-patient learning, patient-centered communication, competency-based education, adaptive learning, and AI governance [1,2,4,5,6,7,8,9,10,11]. First, simulation can facilitate deliberate practice when learning goals, observable performance, and feedback are clearly specified [1,2]. Second, virtual patients offer a controlled setting for information collection, decision-making, and clinical reasoning [4]. Third, patient-centered communication and empathy are observable behaviors that include asking about concerns, recognizing emotions, providing options, and assisting in shared decision-making [7,8]. Fourth, competency-based education must have a traceable relationship between observable performance and defined competencies [9,11]. Fifth, adaptive learning can adjust challenge and feedback while preserving competency standards defined externally by educators or validated frameworks [11,14]. Finally, AI governance must be safe, transparent, accountable, and human-controlled [10,15,16].

These principles form a learning cycle in which learner behavior shapes the simulated encounter, the encounter generates observable performance, performance is interpreted against defined competencies, and feedback informs subsequent learning.

### 3.2. Simulation and Deliberate Practice

Simulation is educationally valuable when its design supports defined learning objectives, deliberate practice, feedback, and progression rather than technological realism alone [1,2]. Accordingly, conversational realism in AI-based simulation should serve identifiable educational purposes by creating opportunities to demonstrate observable competencies.

The proposed architecture therefore treats the clinical encounter as the unit of practice, linking learner interaction, performance evidence, feedback, and subsequent scenario progression. Additional technological complexity is justified only when it supports these educational functions.

### 3.3. Virtual Patients and Clinical Reasoning

Existing virtual-patient approaches vary in case design, interaction, outcomes, and implementation. However, virtual patients provide opportunities to practice information gathering, decision-making, and clinical reasoning in a controlled environment [4].

AI virtual patients need to be consistent throughout the encounter. The simulated patient should be able to keep the relevant clinical information, previous disclosures, unresolved concerns, and interactional context for later responses to ensure that the responses are consistent with the developing case. This is not a claim that the current systems already have the proposed state architecture, but rather a synthesis-derived design requirement.

### 3.4. Generative AI and Conversational Learning

Generative AI and large language models facilitate a wide range of interactions, content generation, custom learning experiences, and assessment-related capabilities [5,6,17,18,19]. However, the current evidence is still inconsistent and cannot confirm that AI-driven interactions can be a substitute for teachers or real clinical practice [5,17,19].

The main design implication is bounded generation. Validated scenario information, the active simulated-patient state, learning objectives, competency criteria, and clinical safety rules should constrain AI-generated responses. If there is model uncertainty that may have a significant effect on the educational or clinical significance of an encounter, then constraints and human review should be prioritized over unconstrained generation.

### 3.5. Practicing Difficult Conversations

Patient-centered communication involves actively asking about concerns and preferences, listening to patients, providing clear information, acknowledging emotions, and encouraging shared decision-making [7]. These behaviors provide concrete objectives for communication training, and evidence from virtual-patient approaches suggests that they may offer opportunities to practice empathy-related behaviors, although evidence of durable gains in learners’ empathy or in real-patient communication remains limited [8].

The proposed architecture does not consider communication as a mere conversation, but as a consequential behavior. Learner questions, explanations, emotional acknowledgment and communication style may affect the simulated disclosure of information, cooperation or scenario progression. This is to support the practice of communication skills, not to suggest that simulated affect is a simulation of human affect. It is not yet proven that the use of AI simulation enhances communication or empathy results; the proposed architecture provides a basis for testing these outcomes, but does not establish that they have improved.

### 3.6. Competency and Adaptive Learning

Interpretation of performance in relation to defined, context-dependent competencies is a key requirement of competency-based education [9]. The proposed framework thus differentiates between three levels of representation:

Learner behavior → behavioral indicator → competency judgment.

For instance, during a medication adherence encounter, asking about the complexity of the medication regimen, beliefs, cost, or other barriers may be behavioral evidence of patient-centered information gathering. The indicator can then be used to assess a defined communication or clinical-management competency. The transcript is not considered proof of competence without contextual interpretation.

Competency standards and assessment criteria should be developed by educators or validated competency frameworks, not by the AI system [9,11].

### 3.7. Assessment and Learning Analytics

The learning pathway should be adjusted to accommodate adaptive learning, not the competency standard. Learning analytics can aid adaptation by transforming interaction data into information about performance trajectories, but the length of the response, the number of words, or individual keywords are not good proxies for competence [11]. Assessment should be based on predetermined behavioral indicators that are associated with specific competencies. Automated assessment can lead to false precision; the handling of uncertainty is specified in Section 4.4.

### 3.8. Safety and Governance

The use of AI in healthcare training comes with potential risks such as inaccuracies or inconsistencies in the content, bias, privacy issues, automation bias, and the potential for overreliance on AI-generated information [10,20,21,22,23,24]. Governance mechanisms for these risks should be incorporated into the architecture rather than treated solely as external considerations.

The framework therefore proposes expert-reviewed scenario specifications, generation boundaries, data minimization, auditability, fairness testing, escalation rules, and human oversight as design safeguards; their implementation and effectiveness still require empirical evaluation. High-stakes assessment, certification, progression and remediation decisions are subject to qualified educator and institutional authority until system reliability and validity are empirically established [10].

### 3.9. Design Requirements

The synthesis across the six evidence domains yields seven proposed design requirements for an AI-based adaptive clinical simulation environment. These requirements are translated into architectural functions through recurring educational needs, technological capabilities, and governance conditions. The requirements, their educational rationale, the literature that supports them, and the design response are summarized in Table 1.

The requirements are not given as independently validated features. Instead, they are the design basis that has been synthesized and on which the proposed architecture is built. The evidence sources and the difference between evidence from the literature, synthesis inference, and author-proposed implementation are explicitly stated in the following evidence-to-design traceability analysis.

The seven requirements are not implemented in isolation. They are coordinated through an integrated adaptive learning loop (Section 4). The need for such coordination is a synthesis-derived inference from the cross-domain evidence [1,2,4,5,6,7,8,9,10,11,14], whereas its specific 11-stage organization is an author-proposed design decision.

## 4. Proposed Framework Architecture

### 4.1. Conceptual Definition

**Figure 1:**
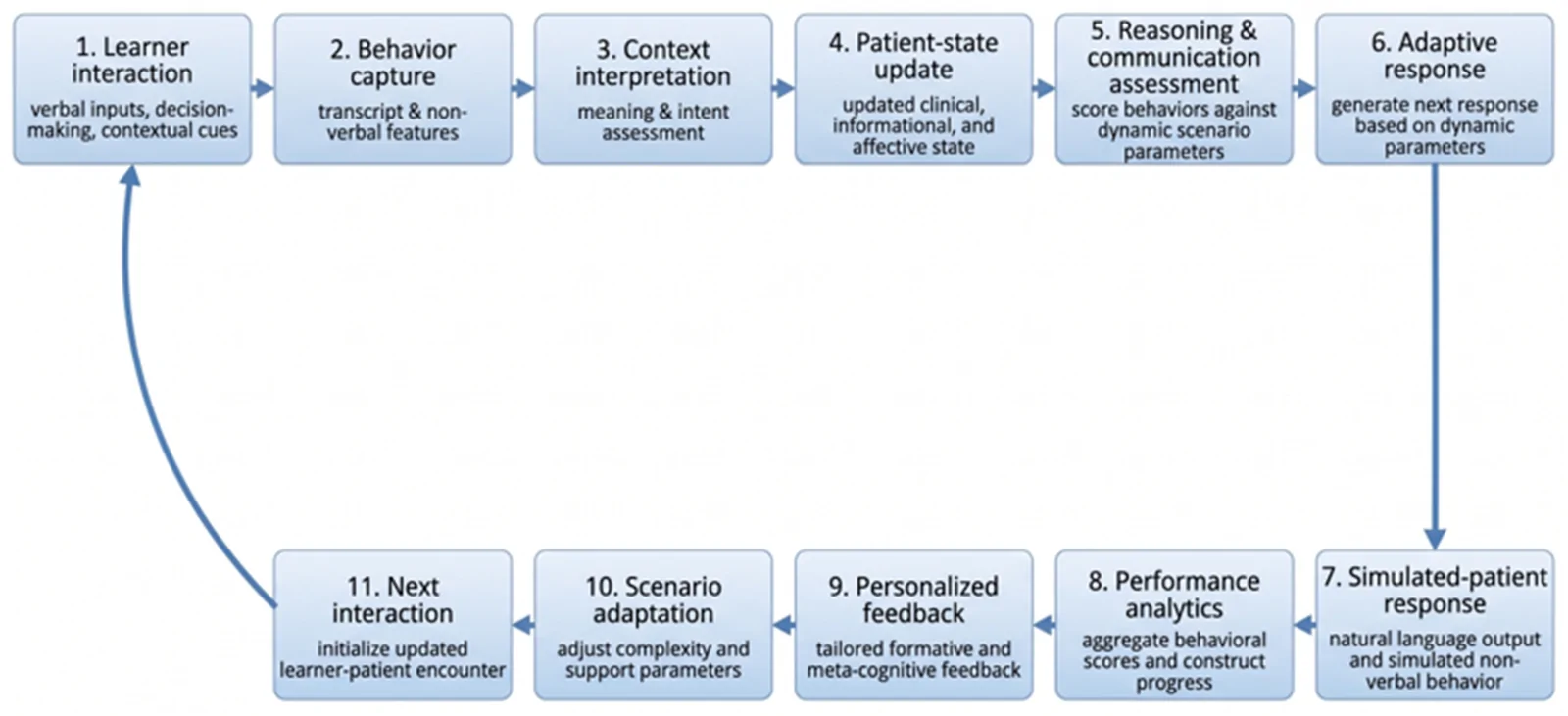
Closed-loop architecture for AI-based clinical simulation and health communication.

The proposed framework is an evidence-informed conceptual architecture that integrates adaptive clinical simulation, patient-centered communication, competency-oriented assessment, and formative feedback within an 11-stage closed loop (Figure 1). Unlike a fixed branching case, the proposed system allows learner behavior to influence the simulated-patient state, subsequent information disclosure, assessment, and learning pathway.

AI supports interpretation, response generation, analytics, and bounded adaptation. Educators and institutions retain authority over competency standards, clinical guardrails, escalation, high-stakes assessment, and progression.

### 4.2. Dynamic Simulated-Patient State

The simulated patient is represented by four dimensions of state, which are connected:

Clinical state: symptoms, findings, relevant history, disease course, and scenario-defined clinical facts.

Informational state: what the patient knows, remembers, has disclosed, or is permitted to disclose at this point in the encounter.

Affective state: scenario-defined emotional cues, e.g., fear, frustration, uncertainty, reassurance.

Interactional state: rapport, trust, unresolved concerns, prior learner responses, and the trajectory of the interaction.

The system reads the learner’s behavior and the state of the system at every interaction and applies the rules of the scenario. A state variable is only changed if a scenario rule or event is defined to support the change. For instance, a well-asked question that is phrased in a relevant way can elicit a symptom that has not been mentioned before, and dismissing a concern can change the interactional state and the simulated patient’s willingness to cooperate. Clinical facts should be consistent with the case specification, and affective changes should be within the boundaries of the scenarios.

State updates are carried over the encounter and feed into patient responses and assessments. The four dimensions can affect each other, but any change must be traceable to the learner’s behavior, a scenario event, or an approved rule. These states are not intended to be a representation of a real patient’s internal psychological processes, but rather an educational tool.

*Representation.* Each state dimension is held as a structured record that the language model can read but cannot overwrite. The clinical state stores case-specification facts (for example, symptom, onset, and severity) as fixed values. The informational state stores, for each fact, a disclosure status (undisclosed or disclosed), the learner behaviors that unlock it, and the turn at which it was disclosed. The affective and interactional states are ordinal variables (for example, low, moderate, or high), each with scenario-defined bounds, together with a list of unresolved concerns and a turn-by-turn interaction log. Only disclosure flags and the affective and interactional variables can change during an encounter; clinical facts cannot.

*Context interpretation and transition rules.* In each turn, context interpretation classifies the learner’s input into predefined behavior types (for example, open question about barriers, acknowledgement of emotion, premature advice, or dismissal of a concern) using the case specification, the encounter history, and the current state, and attaches a confidence value. It does not infer unobserved learner intent. Transition rules are educator-authored condition–action pairs: if a behavior of a given type is detected at or above a confidence threshold, a specified change is applied (for example, unlocking a fact, or raising or lowering an ordinal variable by one step) within the variable’s bounds. The weight of a learner action is expressed by the size of the change its rule permits, and no variable moves more than one step per turn, so that a single action cannot reverse the encounter.

*Conflicting inputs and uncertainty.* When one turn triggers rules with opposing effects (for example, acknowledging an emotion while dismissing a concern), the educator-specified precedence applies, and clinical-consistency and safety rules take precedence over affective and interactional rules. Where no precedence is specified, or when classification confidence is below the threshold, no state variable changes, the patient responds neutrally or asks the learner to clarify, and the turn is flagged for review. Before any generated response is delivered, it is checked against the clinical facts and disclosure flags; a response that contradicts them is blocked and replaced by a scripted fallback, and the deviation is logged (Section 8.4).

*Worked example.* In a medication-adherence case, a cost barrier is initially undisclosed (informational state), and trust is moderate (interactional state). The learner asks, “Is it ever difficult to afford your tablets?” The turn is classified as an open question about barriers (with high confidence, above an educator-set threshold), so the cost barrier is disclosed, and trust rises one step. The learner then says, “You just need to take them as prescribed.” This is classified as dismissal of a concern, so trust falls one step and the patient gives a brief, guarded reply with no new disclosure. A later turn (“So how are things?”) is classified with low confidence, below the threshold, so no state changes and the patient asks what the learner would like to know. Threshold and step values would need to be set empirically (Section 7.1).

### 4.3. Integrated Reasoning and Communication

Clinical reasoning and communication are assessed together because the information available for clinical interpretation can depend on both the learner’s questions and how those questions are asked. The system therefore captures observable verbal, decision-making, and interactional behaviors in the context of the case.

Assessment follows the behavior → indicator → competency sequence defined in Section 3.6; Section 4.5 specifies how indicator evidence is converted into competency judgments.

### 4.4. Adaptive Response and Scenario Progression

Adaptation operates at three levels: interaction, scenario, and learning pathway. At the interaction level, the simulated patient responds according to learner behavior and the current state. At the scenario level, information availability, complexity, or scaffolding may change within predefined case rules. At the learning-pathway level, subsequent practice can target competencies for which performance evidence indicates a learning need.

Repeated evidence of satisfactory performance may justify increased complexity, whereas recurrent errors may prompt structured hints, targeted practice, or additional feedback. As an illustrative default, complexity increases only after satisfactory performance on the relevant indicators across at least three consecutive encounters, and support increases after recurrent errors on the same indicator across at least two encounters; judgments below the confidence threshold or flagged for review count toward neither rule.

Assessment uncertainty is a clear protection. If there is not enough evidence, conflicting evidence, or evidence that may be misinterpreted, the system should ask for additional evidence or mark the assessment for educator review. A low-confidence judgment should not automatically lead to a penalty to a learner or to progression, certification, or remediation. Teachers need to be able to see the evidence and to override any inappropriate adaptations. Judgments of low confidence, disputed, or unreviewed should not be used to make adaptive decisions until the evidence has been reviewed or enough additional evidence has been gathered.

### 4.5. Assessment and Feedback

Assessment combines observable behavior, contextual interpretation, predefined indicators, and competency criteria. Feedback should identify the observed behavior, explain its relevance to the learning objective, and recommend a specific improvement. For example, feedback may identify a missed adherence barrier and recommend exploring the patient’s practical constraints before discussing treatment options.

Assessment thresholds and rubric criteria should be defined before deployment. Their validity, reliability, and agreement with qualified human assessors must be evaluated empirically.

*Producing a competency judgment.* An analytic rubric specifies, for each competency, the indicators, descriptors for each performance level, and examples of qualifying and non-qualifying behavior. For each indicator, the AI component returns an ordinal rating, the supporting transcript excerpts, and a confidence value. Indicator ratings are aggregated into a competency judgment by an educator-defined rule (for example, a minimum number of indicators at the expected level and no critical indicator missed), and cut scores are set through a documented standard-setting procedure with expert panels, not by the model.

*Validity evidence.* Validity evidence would be generated against an explicit interpretation–use argument, organized around the scoring, generalization, extrapolation, and implications inferences of Kane’s framework [25,26]. Scoring evidence is the agreement between AI-supported and expert ratings; generalization evidence is the stability of scores across scenarios, raters, and occasions; extrapolation evidence is the relationship with independently assessed performance, such as standardized-patient or workplace-based ratings; and implications evidence concerns the consequences of feedback, standard setting, and decisions.

*Reference standard, agreement, and calibration.* The reference standard would be the consensus of at least two trained expert raters who are blinded to the AI output. Human–human agreement is established first, because AI–expert agreement cannot be interpreted beyond the reliability of the experts themselves. Agreement is reported with confidence intervals, using intraclass correlation coefficients for continuous scores and weighted kappa for ordinal ratings [27]. AI confidence values are then calibrated against expert ratings on a held-out set: judgments reported with high confidence should agree with the experts at least as well as expert raters agree with one another, and the confidence threshold for automated use rather than educator review is set to achieve this. Calibration is repeated when scenarios, languages, or learner populations change.

*Cultural and linguistic variation.* Rubrics describe functional behaviors (for example, acknowledging a stated concern) rather than specific phrases, and expert panels include raters familiar with the learners’ languages and communication norms. Agreement and score distributions are compared across subgroups defined by first language, accent or dialect (where speech is transcribed), and cultural background. Systematic differences trigger rubric revision, and atypical but functionally appropriate expressions are referred for human review rather than scored as absent.

Feedback remains formative until the relevant assessment process has demonstrated adequate validity and reliability for its intended use. Educators must be able to inspect the supporting evidence, correct inappropriate interpretations, and determine when further teaching or remediation is needed [11].

Table 2 operationalizes the 11 stages by specifying their principal inputs, actions, outputs, and control boundaries.

## 5. Applications in Professional Healthcare Training

The proposed architecture can be applied across clinical history-taking, clinical reasoning, patient-centered communication, shared decision-making, interprofessional training, and competency-oriented assessment. The scenarios below are idealized illustrations of how the architecture is intended to operate; they do not represent implemented or validated interventions, and Section 5.6.2 considers how they may fail.

### 5.1. Clinical History-Taking

Clinical history-taking can be represented as a context-dependent process of information gathering rather than a fixed sequence of questions. The simulated patient’s disclosures are based on the scenario specification, the information already obtained, and the relevance of the learner’s questions. Assessment can then take into account whether relevant information was gathered, how questions were prioritized, whether important cues were identified, and whether communication facilitated effective information gathering.

For instance, in a simulated scenario of undifferentiated abdominal pain, the relevant symptoms can be revealed in response to the right questions, but not in a fixed sequence. Observable behaviors can be linked to indicators of relevant history, clinically important cues, and follow-up questions. Feedback can then be directed at information that was not covered, or questions that were not effective, and subsequent practice can be directed at the learner’s identified needs. The clinical facts and disclosure rules should be consistent with the educator-defined case.

### 5.2. Clinical Reasoning

Clinical reasoning is a process of gathering information, generating hypotheses, interpreting, prioritizing, and making decisions, which is iterative. The proposed architecture can enable practice by enabling the simulated encounter to react to the learner’s actions within a set of clinical boundaries. New information, changing priorities of the scenarios, or missing cues may cause the learner to re-evaluate an interpretation or re-think a decision.

The educational emphasis is not only on whether the learner achieves the desired answer, but whether the process of thinking is suitable to the information that is available at that stage of the encounter. Feedback can highlight missed cues, unsupported conclusions, premature closure, or lack of reassessment. This practice can be used in addition to supervised clinical exposure but cannot be taken for granted to be a substitute for authentic clinical experience or to prove that it enhances clinical performance without empirical testing.

### 5.3. Communication and Empathy

Simulated encounters involving serious diagnoses, uncertainty, anger, fear, treatment concerns, or distress can provide structured opportunities to practice difficult communication. Within the proposed architecture, scenario-defined affective and interactional states may change in response to observable learner behaviors, such as acknowledging concerns, explaining information clearly, responding to emotion, or dismissing a patient’s perspective.

Assessment should focus on specified behaviors—including acknowledgment of concerns, clarity, responsiveness, and appropriate explanation—rather than conversational fluency alone. Feedback can identify which behaviors were demonstrated, which were missed, and how the interaction might be improved. However, successful use of empathic language in a simulation does not establish that a learner has developed genuine empathy or will demonstrate equivalent behavior with real patients. The proposed application therefore supports structured practice and behavior-based feedback, not an unqualified claim about emotional development [8]. Direct evidence that AI-driven simulation enhances learners’ communication or empathic competence, as distinct from providing opportunities for rehearsal and receiving feedback, remains limited and heterogeneous [5,8]; such outcomes are therefore treated here as hypotheses to be evaluated (Section 7), not as expected benefits.

### 5.4. Shared Decision-Making

Shared decision-making involves the clinician asking the patient about his or her values, preferences, concerns, understanding, and treatment priorities, and then including these in a shared plan. These elements can be simulated in the informational and interactional state of the simulated patient, allowing for the presentation of relevant preferences during the encounter, rather than as a standalone checklist.

Learners can practice asking for preferences, giving a balanced explanation of options, expressing uncertainty, checking for understanding, and creating a plan that incorporates the priorities of the simulated patient. These observable behaviors can be mapped to shared decision-making competencies that have been defined. Feedback can help determine, for instance, if the learner described alternatives but did not establish the patient’s priorities before suggesting a course of action for the patient.

### 5.5. Interprofessional and Scenario-Based Training

The architecture can be tailored to various professional roles and complex scenarios such as medication adherence, counseling, conflict management, multimorbidity, and interprofessional communication. Competing priorities, incomplete information, multiple stakeholders, and changing clinical circumstances can be used to increase the complexity of the scenarios, while keeping the learning objectives and competency criteria explicit.

The scenario may include role-specific responsibilities, information-sharing needs, and opportunities for collaborative planning for interprofessional training. Assessment may include an evaluation of how well learners convey relevant information, identify professional boundaries, make decisions, and react to uncertainty. The same architecture can be used for various professions and curricula, but the content of scenarios, assessment rubrics, and governance requirements need to be adapted to the intended setting.

### 5.6. Adaptive Assessment and Feedback

Across these applications, adaptive assessment follows the sequence defined in Section 3.6 (learner behavior → behavioral indicator → competency judgment), followed by feedback or learning adaptation as specified in Section 4.4 and Section 4.5. Criteria, thresholds, and uncertainty handling follow those sections, and the validity and reliability of these procedures remain empirical questions (Section 7).

#### 5.6.1. Illustrative Scenario Applications

The scenarios in Table 3 demonstrate how the proposed architecture can be applied across different competency domains.

The scenarios are illustrative applications of the proposed framework, not validated assessment instruments or evaluated interventions. Competency criteria, state-transition rules, clinical content, and performance thresholds require appropriate definition and review before implementation.

Taken together, these scenarios illustrate how the same architecture can support different educational objectives without requiring a different underlying framework for each scenario. Implementation and evaluation are required to determine whether the resulting interactions, assessments, and adaptations are sufficiently consistent, safe, valid, and educationally useful.

#### 5.6.2. Failure Cases and Boundary Conditions

The scenarios above describe the correct operation. They are analytical projections from the proposed design logic, not observed failures, and the corresponding risks, monitoring, and escalation are mapped to stages in Section 8.4. The following cases describe the possible failure of the same encounters, detection of each failure, and the limitation of the framework’s educational consequences.

- **Inconsistent state evolution (abdominal pain).** The patient says that the pain started yesterday and, later, that it started a week ago, or discloses a symptom absent from the case specification. The consistency check (Section 4.2) should block the response and substitute a scripted fallback; the deviation is logged, the affected turns are excluded from assessment so that the learner is not penalized, and the case is withdrawn for correction if the failure recurs. If the check itself fails, the learner may receive misleading information, which is why post-session log review and educator invalidation of encounters are retained.
- **Ambiguous behavior and conflicting signals (new cancer diagnosis).** A learner provides an empathic statement but immediately follows it with premature reassurance or asks a question that could be genuine exploration or a stock phrase. Rules may then conflict, or confidence may fall below the threshold. Precedence rules are applied or, failing that, no state change is made and the turn is flagged. Feedback reflects both behaviors, and any indicator lacking evidence is recorded as “insufficient evidence”, rather than “absent”.
- **Inaccurate competency judgments (diabetes treatment decision).** The AI component credits a formulaic phrase as preference elicitation or fails to credit an appropriate expression in another dialect or communication style, overstating or understating competence. Evidence excerpts and confidence values are shown to educators, a sample of judgments is audited against expert ratings, subgroup agreement is monitored (Section 4.5), and judgments remain formative until agreement targets are met.
- **Hallucination.** The language model introduces a medication, test result, or piece of advice that is not in the case specification [20,22,23]. Bounded generation and the pre-delivery consistency check are the first defense, and critical violations suspend the scenario for review (Section 8.4). These controls cannot eliminate hallucination, which is why unsupervised high-stakes use is excluded.
- **Propagation into adaptation.** An erroneous or low-confidence judgment could make a scenario inappropriately easy or difficult. Because adaptation requires consistent evidence across encounters and excludes flagged judgments (Section 4.4), a single error should not change the pathway, but a systematic error (for example, a rubric that mis-scores a subgroup) could; educator override and periodic audits are the corresponding safeguards.

The frequency, detectability, and educational impact of these failures are empirical questions for the technical and expert validation stages (Section 7.1).

## 6. Comparison with Existing Approaches

### 6.1. Comparative Positioning

Existing approaches provide important foundations for AI-supported healthcare training, but they differ in their principal educational and technological emphases. Conventional virtual patients provide structured clinical cases and opportunities to practice information gathering and clinical reasoning [4]. Generative-AI-supported virtual patients can enable more flexible, open-ended dialog [5,6]. Affective computing contributes approaches for representing or responding to emotion-related cues, while adaptive learning systems support learner-specific practice and progression. AI-supported assessment and learning analytics can help organize performance evidence and provide feedback [8,11]. Intelligent tutoring systems add an explicit learner model, step-level feedback, and task selection, with meta-analytic evidence of positive learning effects relative to conventional instruction [28,29]; AI-tutored simulation improved procedural skill acquisition in a randomized trial [30]; language-model-powered simulated patients with automated feedback are feasible for history-taking, with high but not-uniform agreement between model and human ratings [31]; and serious games and gamified simulations show effects at least comparable to conventional teaching, although mostly from low-quality evidence [32].

These approaches add complementary capabilities, but the presence of one capability does not necessarily mean that it is integrated with the other aspects of an educational encounter. For instance, conversational flexibility does not guarantee continuity of simulated-patient state, emotion-related responses do not necessarily imply valid assessment of communication competence, and adaptive progression does not imply that decisions are based on reliable competence evidence.

To make the comparison explicit, the approaches in Table 4 are compared on five dimensions: (i) persistent, learner-responsive state in the task environment; (ii) interpretation of open-ended learner input, including communication; (iii) mapping of performance to predefined competencies through documented indicators; (iv) adaptation that depends on that evidence, with explicit uncertainty handling; and (v) governance and human override specified at the level of individual stages. Intelligent tutoring systems are the closest precedent, typically combining step-level feedback with task selection from a learner model. In the proposed architecture, stages 3, 5, and 9 correspond to step-level assessment and feedback, stage 8 to the learner model, and stage 10 to task selection. The difference lies in stages 4, 6, and 7: the task environment is itself a stateful interlocutor whose disclosures and cooperation depend on the learner’s communication, so a patient model and a learner model are coupled and updated in the same cycle. Among the sources reviewed, no intelligent tutoring system, AI-tutored simulation, or language-model simulated patient was identified that implemented all five dimensions together. This reflects the limits of the evidence base (Section 2.2) and should not be interpreted as the outcome of a systematic inventory.

The proposed architecture addresses this integration challenge by coordinating learner interaction, persistent simulated-patient state, clinical reasoning and communication assessment, adaptive response, performance analytics, feedback, and scenario progression within an explicit 11-stage closed loop. Its distinguishing feature is therefore not the invention of a new AI capability, but the proposed organization of complementary capabilities around an evolving simulated encounter and an educator-governed competency model.

Table 4 compares the principal emphases of these approach categories and identifies the integration role proposed by the framework. The comparison is conceptual rather than the result of a systematic inventory of all available platforms. It should not be interpreted as evidence that every existing system lacks integrated functionality or that the proposed architecture has demonstrated superior performance.

### 6.2. Novelty and Contributions

The conceptual contribution of this article is in four related design propositions.

First, it is suggested that the organizing mechanism between the clinical facts, information revealed, scenario-defined affective cues, and interactional history of an encounter is a persistent simulated-patient state. This can be used as a foundation for continuity between learner actions and subsequent simulated responses.

Second, clinical reasoning and patient-centered communication are considered as interrelated aspects of performance. The information that the learner receives may be affected by the relevance and the way the learner is being questioned, and the communication behaviors may affect the next interaction. Their evaluation, however, is still based on clear learning goals and measurable actions.

Third, there is a competency-oriented feedback loop between assessment and adaptation. Learner behavior is interpreted in relation to predefined indicators and competency criteria, and the evidence can be used to provide formative feedback and further practice. Judgments of uncertainty or consequence will continue to be subject to educator review.

Fourth, governance is not just a deployment consideration but is part of the proposed architecture. The purpose of clinical guardrails, scenario constraints, uncertainty handling, auditability, and human authority is to influence response generation, assessment, adaptation, and progression.

These propositions outline an integrated design model for future development and evaluation. The framework offers a systematic approach to describing system components, examining their respective roles, and assessing the impact of combining them to enhance the consistency, validity, safety, and educational value of simulated encounters.

Relative to the approaches in Table 4, these propositions contribute a structured synthesis with three distinguishing mechanisms: (1) the coupling of a persistent patient model and a learner model in one cycle; (2) uncertainty-gated state updating, assessment, and adaptation, in which low-confidence evidence leaves the state, the assessment, and the learning pathway unchanged; and (3) failure modes mapped to individual stages with detection, escalation, and human override (Section 8.4). The framework is not a new algorithm or technology, and whether it constitutes an advancement over existing integrated systems is an empirical question that the validation pathway in Section 7 is designed to address.

This is a conceptual comparison of approach categories, not a systematic review of individual products or platforms. The descriptions indicate principal emphases and proposed integration roles rather than universal properties of every system in each category. The integrated architecture has not yet been empirically validated.

## 7. Validation and Research Agenda

**Figure 2:**
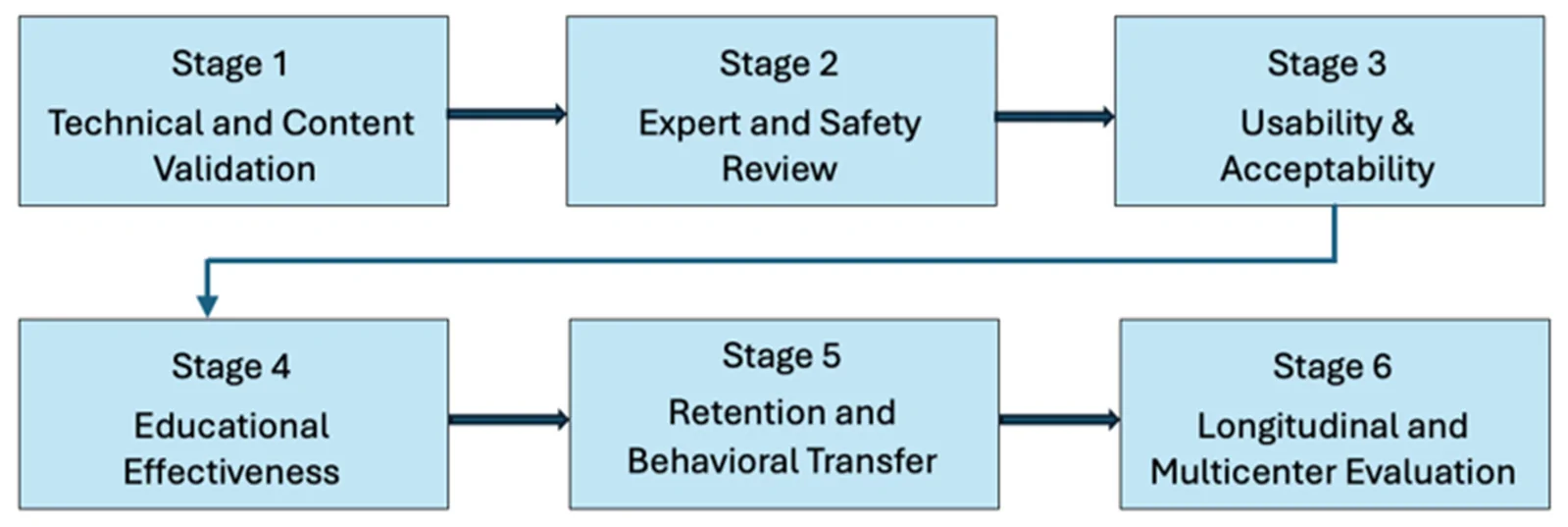
Staged validation pathway for the proposed architecture.

The proposed architecture requires staged evaluation before its technical reliability, educational effectiveness, and potential for clinical transfer can be established. As illustrated in Figure 2, validation should progress from technical and content checks to expert review, usability, educational effectiveness, and longer-term transfer. Progression between stages should depend on predefined criteria appropriate to the intended use. There are three prioritized outcomes which determine whether later, more costly studies are justified: (1) state consistency and safety of generated responses; (2) agreement between AI-supported and expert competency ratings; and (3) competency gain on blinded, independently rated performance relative to an active comparator. Usability, retention, and transfer are evaluated in turn once these are satisfied.

### 7.1. Technical, Content and Safety Validation

Initial testing should assess adherence to predefined patient-state-transition rules, clinical consistency, consistency of information disclosure, and compliance with clinical and educational constraints. Health profession educators and clinical experts should review scenario content, competency mappings, simulated-patient responses, and feedback. Experts’ agreement should be quantified as described in Section 4.5, with the indicative targets in Table 5.

Testing should also look for potential problems, such as unbacked clinical claims, conflicting information, disclosure issues, biased answers, and adaptations that are based on incorrect evaluations. If there are critical failures, revision and retesting should take place prior to learner-facing evaluation. The usability test should measure task completion, cognitive workload, usefulness of feedback, and proper recognition of system limitations. Satisfaction should not be used as a measure of the effectiveness of learning.

Suggested design: a prespecified test suite of scripted learner-turn sequences, including ambiguous, conflicting, and adversarial inputs, run repeatedly across scenarios. For safety-critical events, an observed count of zero in n independent-test turns bounds the true event rate below approximately 3/n with 95% confidence (the “rule of three”), so about 300 turns are needed to exclude a critical-failure rate above 1%.

### 7.2. Comparative and Educational Evaluation

Comparative studies should separate conventional virtual patients from non-adaptive generative-AI encounters from adaptive encounters without the full feedback loop from the proposed integrated architecture where possible. To facilitate meaningful interpretation, learning objectives, scenario content, exposure time, and assessment conditions should be similar.

Outcomes should be based on predefined competencies, including history-taking, clinical reasoning, and communication and shared decision-making. Assessment should be based on explicit rubrics and, if appropriate, blinded human raters. Appropriate evidence of validity and reliability, such as inter-rater agreement if applicable, should be reported. The distinction between usability and perceived usefulness and demonstrated competency gains should be made.

The primary educational comparison should be a randomized or cluster-randomized trial with an active comparator (conventional virtual patient or a non-adaptive generative-AI encounter), and the primary outcome and analysis plan should be prespecified. The primary outcome would be a competency score (e.g., blinded, rater-assessed), and the sample size would be determined by the smallest difference that would be considered educationally meaningful. Effects would be reported with confidence intervals. Secondary outcomes would be process measures, usability and subgroup effects.

### 7.3. Retention and Behavioral Transfer

Studies should assess baseline and post-training performance and, where feasible, retention at follow-up at a prespecified interval (for example, three months). Transfer should be examined using unfamiliar scenarios, standardized-patient encounters, or other independently assessed tasks. Workplace-based and patient-level outcomes should be investigated only when justified by preceding evidence and suitable study designs.

Longitudinal and multicenter studies should examine whether findings generalize across learner groups, professional roles, languages, and institutional settings. Table 5 summarizes the proposed validation stages, designs, and indicative benchmarks.

The stages, designs, and benchmarks are indicative proposals for future evaluation, not results or universal acceptance thresholds; criteria should be prespecified and justified for each intended application, and critical safety requirements should be met before progression to learner-facing evaluation.

## 8. Ethical, Safety, and Governance Considerations

Safety and governance must be embedded throughout the proposed architecture rather than treated as separate implementation concerns. The principal safeguards concern clinical accuracy, fairness, privacy, and human accountability.

### 8.1. Clinical Accuracy and Reliability

The information provided in the AI-generated responses may be inaccurate, contradictory, or unsupported. Therefore, scenarios should be based on clinical content that has been reviewed by experts, bounded response rules, and predefined escalation conditions. The tests should include checks for the consistency of scenarios, inappropriate disclosure of information, and violations of clinical or educational constraints. If there are any critical failures, they should be logged and reviewed before further use.

Educators need to have the discretion to edit, correct, and override inappropriate outputs [10,11,20,21,22,23,33,34,35].

### 8.2. Bias and Cultural Responsiveness

Demographic, linguistic or socioeconomic biases may be represented in simulated-patient responses and assessment criteria. Potential unfairness should be identified using diverse scenario profiles, expert review, and testing across relevant communication styles. Assessment should be based on the intended competency and its clinical application, not on linguistic or cultural differences that do not affect the competency [11,36,37].

### 8.3. Privacy and Learner Data

Learner interaction logs may reveal clinical reasoning, errors, communication patterns, and performance. Data collection should follow purpose limitation and minimization principles. Learners should be informed about data collection, analytical use, access, retention, and any role in formal assessment. Institutions should establish appropriate access controls, secure storage, retention policies, and clear separation between formative learning records and high-stakes assessment records.

### 8.4. Human Oversight and Accountability

Educators and institutions should have control over the content of scenarios, competency standards, safety limits, escalation, remediation, and consequential decisions. The system should provide access to the relevant assessment evidence and uncertainty for review, keep an audit trail of outputs and resulting adaptations, and allow for human intervention where assessments differ from expert judgment or where cases are outside of the boundaries.

Table 6 shows the main risks associated with the various stages of the architecture, monitoring approaches, safeguards, and accountable human oversight. These are proposed governance needs and need to be evaluated in the context of implementation.

## 9. Discussion

### 9.1. Principal Contribution

The primary contribution of the framework is not about proposing a new AI capability; instead, it is an integrated conceptual architecture. The integrated architecture provides a common structure for designing and evaluating capabilities that are often addressed separately.

The framework also shifts evaluation beyond conversational realism toward clinically coherent interactions, observable competency development, appropriate adaptation, feedback quality, and safety. These proposed benefits require empirical validation.

### 9.2. Educational Implications

The framework could expand access to repeated practice of encounters that are not easy to schedule regularly in clinical placements. Linking observable learner behavior to competency-based feedback may also help educators identify learning needs and plan targeted remediation.

Its intended role is complementary to clinical placements, standardized-patient encounters, existing simulation, and educator-led teaching. Its potential contribution lies in integrating history-taking, reasoning, communication, shared decision-making, and reflection within a single simulated encounter.

### 9.3. Relationship to Patient-Centered Care

The relationship between AI-based training and patient-centered care is indirect and prospective. Training may develop competencies relevant to professional practice, but improved simulated performance does not establish better patient experiences, safer clinical decisions, or improved patient outcomes. The same caution applies to learners’ communication and empathy: evidence that AI-driven simulation improves these competencies, rather than offering a means of practicing and measuring them, remains limited [5,8].

Accordingly, patient-care benefits should be treated as hypotheses for future research, not demonstrated effects of the proposed architecture.

### 9.4. Limitations

This article is a structured narrative review and conceptual synthesis rather than a systematic review with formal risk-of-bias assessment. The evidence is heterogeneous, especially for generative-AI-based virtual patients, and some studies focus on short-term or selected educational outcomes [5,6,38].

The literature search and selection process has further limitations. The search was iterative and domain-based rather than protocol-driven and relied mainly on PubMed together with publisher and organizational sources; relevant work indexed elsewhere may therefore have been missed. As described in Section 2.2, search dates, complete search strings, and record counts were not recorded, and no screening flow diagram was produced. Searching, selection, and categorization of the evidence were carried out by one author, without independent duplicate screening or a formal procedure for resolving disagreements, and sources were retained for conceptual relevance and design utility rather than exhaustive coverage. The evidence base may therefore reflect selection bias, and the provenance categories assigned in Table 1 represent the authors’ judgment. A prospectively documented search and selection protocol would be needed to replicate or extend this synthesis.

There has been no implementation or empirical validation of the framework as an integrated system. The proposed state-transition rules, competency mappings, adaptive decisions, and feedback mechanisms are thus in need of operational testing. Simulated affect is not real empathy, and AI interpretation can differ between languages, cultures, disabilities, and interaction styles. The operational parameters in Section 4.2 and Section 4.4 (thresholds, step sizes, and encounter counts), the failure cases in Section 5.6.2, and the benchmarks in Table 5 are illustrative examples proposed by the authors based on the design logic and the literature cited and have not been tested; the failure analysis is analytical, not empirical. The comparison of intelligent tutoring to adaptive-simulation systems in Section 6.1 is based on selected sources, and not on a list of platforms.

Finally, the framework is technology-agnostic. This helps with possible application in various technical contexts, but leaves the implementation options, integration needs, and performance in the context to future investigation.

### 9.5. Future Research

Future research should operationalize the architecture and evaluate it through the staged validation pathway in Figure 2 and Table 5. Priority should be given to the technical-safety, assessment-agreement, and educational-effectiveness outcomes identified in Section 7.

Research should also examine cultural and linguistic responsiveness, accessibility, educator workload, privacy, and governance. This staged approach would help distinguish technical feasibility from educational effectiveness and educational effectiveness from clinical benefit.

## 10. Conclusions

This article presents an evidence-informed conceptual framework for AI-based adaptive clinical simulation and health communication in professional healthcare training. By integrating persistent simulated-patient state, clinical reasoning, patient-centered communication, competency-oriented assessment, adaptive feedback, and human governance within an 11-stage learning loop, the framework provides a structured basis for designing and evaluating integrated AI-supported learning environments.

The framework is a conceptual proposal, not a validated educational intervention. Its technical reliability, assessment quality, safety, educational effectiveness, and potential transfer to clinical practice must be established through staged empirical evaluation. By defining the relationships among learner behavior, simulated-patient responses, competency assessment, and adaptive feedback, the framework offers a foundation for future implementation and research without presuming improved patient outcomes.

## Figures and Tables

**Table 1:** Evidence-informed design requirements and evidence-to-design traceability for AI-based clinical simulation and health communication.

Proposed Design Response	Evidence Basis and Provenance	Supporting References	Educational Need	Design Requirement
Maintain scenario facts, encounter history, and relevant learner actions across interactions.	The literature on virtual-patient learning and clinically grounded AI Requirement: synthesis derived.	[4,21]	Clinically coherent reasoning and communication	R1. Context awareness
Represent and update clinical, informational, affective, and interactional states.	The literature on virtual patients, generative-AI-supported simulation, and empathy training Requirement: synthesis derived.	[4,5,8]	Continuity and consequential learner–patient interaction	R2. Dynamic patient state
Link simulated-patient disclosure and interactional responses to learner communication.	The literature on patient-centered communication and empathy-related behaviors Requirement: literature derived.	[7,8]	Practice of patient-centered communication and empathy	R3. Responsive communication
Adjust scenario difficulty, scaffolding, and practice according to assessed learning needs, within predefined educational boundaries.	The literature on AI-supported educational assessment and adaptive learning Requirement: literature derived.	[11,14]	Individualized challenge and targeted practice	R4. Adaptive learning
Map observable learner behaviors to predefined performance indicators and competency criteria.	The literature on competency-based education and assessment principles Requirement: literature derived.	[9,11]	Meaningful, defensible evaluation of performance	R5. Competency-oriented assessment
Provide behavior-linked formative feedback and track performance over time.	The literature on simulation-based learning, feedback, and educational assessment Requirement: literature derived.	[1,2,11]	Actionable improvement and monitored learning progression	R6. Feedback and learning analytics
Apply constrained generation, monitoring, auditability, data protection, safety escalation, and human oversight.	Guidance and research concerning AI governance, model risks, and reliability Requirement: literature derived.	[10,15,16,22,24]	Clinical and educational trustworthiness	R7. Safety and governance

Note: R1–R7 denote the seven design requirements. Provenance categories follow Section 2.3: literature derived (directly supported by the cited sources), synthesis derived (inferred by integrating evidence across the six domains), and author proposed (design decisions introduced by the authors). Requirement labels indicate the provenance of the underlying need; the organization of the requirements and all design responses are author proposed.

**Table 2:** Operational stages of the proposed closed-loop architecture.

Control Boundary	Output	Input and Operation	Stage
Learner action.	Interaction or decision data.	Learner asks questions, explains, or makes decisions.	1. Learner interaction
AI-supported extraction; preserve source evidence.	Structured behavior record.	Capture relevant verbal, decision-making, and interactional behaviors.	2. Behavior capture
Scenario rules and learning objectives constrain interpretation.	Contextualized behavior interpretation.	Interpret behavior against case facts, encounter history, and learning objectives.	3. Context interpretation
Case consistency and predefined transition rules.	Updated, persistent state.	Apply authorized update rules to clinical, informational, affective, and interactional states.	4. Patient-state update
Educator-defined rubric; uncertainty retained.	Provisional assessment with supporting evidence.	Map contextualized behavior to indicators and competency criteria.	5. Reasoning and communication assessment
Generation constraints and educator-defined rules.	Candidate response or action.	Select a response based on the updated state, learner behavior, and scenario objectives.	6. Adaptive response
Clinical guardrails and bounded generation.	State-consistent patient response.	Generate dialog or behavior consistent with the state and case specification.	7. Simulated-patient response
Do not treat proxies as validated competence.	Performance profile and uncertainty flags.	Aggregate interaction evidence and relevant competency indicators over time.	8. Performance analytics
Educator review when evidence is uncertain or consequential.	Specific formative feedback and next-step recommendation.	Compare observed behavior with defined criteria and identify learning needs.	9. Personalized feedback
Curriculum rules; no autonomous high-stakes progression.	Revised learning opportunity.	Adjust difficulty, scaffolding, or targeted practice according to supported learning needs.	10. Scenario adaptation
Persistent case rules and educator oversight.	New interaction feeding the next cycle.	Continue with the updated state and selected learning opportunity.	11. Next interaction

**Table 3:** Illustrative applications of the proposed framework across professional healthcare training.

Potential Feedback or Adaptation	Observable Behavior and Competency Link	State or Interactional Change	Scenario
Feedback on missed cues; targeted history-taking or reasoning practice	Relevant history-taking, cue recognition, question prioritization → history-taking and clinical-reasoning competencies	Relevant symptoms are disclosed according to questions and case rules	Undifferentiated abdominal pain
Feedback on specific communication behaviors; additional practice with emotional cues	Acknowledgment of concerns, clarity, responsiveness → difficult-communication competency	Scenario-defined affective and interactional states respond to the learner’s communication	New cancer diagnosis
Feedback on gaps between elicited preferences and the proposed plan	Preference elicitation, balanced explanation, incorporation of priorities → shared decision-making competency	Patient preferences and concerns influence the discussion	Diabetes treatment decision
Targeted de-escalation practice and feedback on responses to concerns	Listening, acknowledgment, de-escalation, professional communication → conflict-management competency	The interactional state changes according to the learner’s response and scenario rules	Angry patient after delayed care
Feedback on overlooked barriers; focused counseling practice	Exploration of barriers, patient-centered questioning, collaborative planning → counseling and information-gathering competencies	Practical barriers and treatment beliefs become available through appropriate inquiry	Medication-adherence problem
Feedback on prioritization and reassessment; appropriately bounded increase in case complexity	Prioritization, uncertainty management, reassessment → integrated clinical-reasoning competency	Competing priorities and relevant clinical information require integration	Complex multimorbidity

**Table 4:** Conceptual comparison of existing approaches with the proposed integrated architecture.

Integration Issue Addressed by the Proposed Architecture	Contribution to Healthcare Training	Principal Emphasis	Approach
Connects encounter history and patient-state updates to subsequent interactions and learning decisions	Supports clinical information gathering, decision-making, and reasoning practice [4]	Structured cases and predefined interaction	Conventional virtual patients
Constrains generated responses using scenario facts, persistent state, learning objectives, and safety rules	Enables more varied conversational interaction and context-sensitive responses [5,6]; automated history-taking feedback showed high but variable agreement with human ratings [31]	Flexible, open-ended dialog	Generative-AI-supported virtual patients
Links scenario-defined affective changes to observable learner communication while avoiding claims that simulated affect reproduces genuine human emotion	Offers approaches for representing or responding to affect-related information	Emotion-related cues and responses	Affective AI
Connects adaptation to competency-relevant evidence, explicit learning objectives, uncertainty handling, and educator oversight	Supports targeted practice and adjustment of learning activities	Learner-specific challenge and progression	Adaptive learning systems
Links behavior to predefined indicators and competencies, then connects assessment to formative feedback and subsequent practice	Supports the organization and interpretation of learner-performance evidence [8,11]	Performance analysis and feedback	AI-supported assessment and learning analytics
Adds a persistent, communication-responsive patient state and uncertainty-gated, educator-governed progression to learner-model-based tutoring	Meta-analytic evidence of positive learning effects relative to conventional instruction [28,29]	Learner model, step-level feedback, and task selection	Intelligent tutoring systems
Extends adaptation from procedural or knowledge tasks to communication-dependent encounters with a stateful patient	Improved procedural skill acquisition in a randomized trial [30]; effects at least comparable to conventional teaching in a systematic review of serious games, mostly from low-quality evidence [32]	Adaptive feedback and challenge in simulated or game-based tasks	AI-tutored and adaptive simulation, including serious games
Provides a common design structure for coordinating these functions under explicit educational and governance constraints	Coordinates interaction, state updating, assessment, adaptation, feedback, and progression within one proposed learning cycle	Persistent simulated-patient state and an explicit competency-feedback loop	Proposed integrated architecture

**Table 5:** Proposed staged validation pathway for the AI-based adaptive clinical simulation architecture.

Decision Consideration	Example Measurable Criteria, Designs, and Indicative Benchmarks	Validation Stage
Revise and retest if critical consistency or safety criteria are not met	Clinical contradiction rate; consistency of information disclosure; proportion of state transitions following predefined rules; frequency of constraint violations; indicative benchmark: no critical contradictions or constraint violations in a prespecified test set of about 300 turns, and at least 95% of state transitions conforming to the rules	Technical and content validation (priority 1)
Resolve material disagreements and critical safety issues before learner testing	Expert ratings of clinical accuracy, response relevance, competency mapping, feedback quality, and safety; inter-rater and AI–expert agreement; indicative target: lower bound of the 95% confidence interval for the intraclass correlation of at least 0.75 (“good” reliability [27]) for formative use and above 0.90 for consequential use, with agreement not materially lower in any prespecified language or background subgroup	Expert and safety review (priority 2)
Address usability barriers and inappropriate reliance on AI	Task completion; cognitive workload; feedback usefulness; trust calibration; recognition of system limitations	Usability and acceptability
Interpret outcomes against prespecified criteria and study limitations	Predefined competency scores; between-group differences; effect estimates and confidence intervals; relevant reliability and validity evidence; design: randomized comparison with an active comparator, prespecified primary outcome rated by blinded assessors, and sample size based on the smallest educationally meaningful difference	Educational effectiveness (priority 3)
Determine whether gains persist and extend beyond practiced scenarios	Follow-up performance; unfamiliar-scenario performance; standardized-patient assessments; independent or blinded ratings; follow-up at a prespecified interval (for example, three months)	Retention and behavioral transfer
Assess generalizability and identify context-specific adaptations	Performance over time; between-site variation; justified subgroup analyses; prespecified margin for between-subgroup differences; longer-term transfer	Longitudinal and multicenter evaluation

**Table 6:** Core governance risks, monitoring and safeguards in the proposed architecture.

Accountable Oversight	Safeguard and Escalation	Detection or Monitoring	Relevant Stages	Risk or Failure Mode
Clinical experts and educators	Constrain generation; suspend or flag critical outputs for review	Clinical consistency checks; expert review; violation logs	Context interpretation; state update; adaptive response	Inaccurate or contradictory clinical output
Scenario designers and clinical educators	Prevent unsupported transitions; log deviations; require correction before reuse	Compare transitions and disclosures with predefined scenario rules	Patient-state update; simulated-patient response	Inappropriate state transition or information disclosure
Educators and institutional governance leads	Revise scenarios or rubrics; investigate systematic disparities	Review across relevant demographic, linguistic, and communication profiles	Response generation; assessment; feedback	Biased responses or assessment
Educators and assessment leads	Avoid automatic penalties or progression under material uncertainty; refer for human review	Review behavior–indicator mappings, uncertainty, and disagreement with human ratings	Assessment; feedback; scenario progression	Incorrect competency assessment or adaptation
Scenario designers and educators	No state change below threshold; apply precedence rules; neutral or clarifying response; flag for review	Classification confidence; rule conflicts; flagged turns reviewed against human coding	Behavior capture; context interpretation; patient-state update	Ambiguous or conflicting learner input; low-confidence classification
Institution and data-governance personnel	Minimize data; restrict access; apply secure retention and deletion policies	Access monitoring; data-use and retention audits	Behavior capture; analytics; record retention	Privacy breach or inappropriate data use
Educators and institutional leadership	Preserve human authority over certification, progression, and remediation	Audit consequential decisions and overrides	Assessment; progression; remediation	Automation bias or high-stakes misuse
Researchers and institutions	Report limitations; require appropriate further evaluation before clinical claims	Examine whether claims are supported by transfer or clinical-outcome evidence	Validation and outcome evaluation	Unsupported claims of clinical benefit

## Data Availability

No new data were created or analyzed in this study. Data sharing is not applicable to this article.
